# Oral Administration of *Lactobacillus* Inhibits the Permeability of Blood-Brain and Gut Barriers in a Parkinsonism Model

**DOI:** 10.1155/2023/6686037

**Published:** 2023-11-09

**Authors:** Angélica Y. Nápoles-Medina, Blanca R. Aguilar-Uscanga, Josué R. Solís-Pacheco, Aldo R. Tejeda-Martínez, Luis J. Ramírez-Jirano, María F. Urmeneta-Ortiz, Veronica Chaparro-Huerta, Mario E. Flores-Soto

**Affiliations:** ^1^Laboratorio de Neurobiología Celular y Molecular, División de Neurociencias, Centro de Investigación Biomédica de Occidente (CIBO), Instituto Mexicano del Seguro Social, Sierra Mojada #800, Independencia Oriente, C.P. 44340 Guadalajara, Jalisco, Mexico; ^2^Departamento de Farmacobiología, Laboratorio de Microbiología Industrial, Centro Universitario de Ciencias Exactas e Ingenierías, Universidad de Guadalajara, Blvd. Marcelino García Barragán # 1421, Olímpica, C.P. 44430 Guadalajara, Jalisco, Mexico; ^3^División de Neurociencias, Centro de Investigación Biomédica Occidente (IMSS), Guadalajara, Mexico

## Abstract

It has recently been shown that the administration of probiotics can modulate the microbiota-gut-brain axis and may have favorable effects in models of Parkinson's disease. In this study, we used a hemiparkinsonism model induced by the neurotoxin 6-OHDA to evaluate the efficacy of the administration of a four-week administration of a mixture containing the microorganisms *Lactobacillus fermentum* LH01, *Lactobacillus reuteri* LH03, and *Lactobacillus plantarum* LH05. The hemiparkinsonism model induced an increase in rotations in the apomorphine test, along with a decrease in the latency time to fall in the rotarod test on days 14 and 21 after surgery, respectively. The administration of probiotics was sufficient to improve this condition. The model also showed a decrease in tyrosine hydroxylase immunoreactivity in the striatum and the number of labeled cells in the substantia nigra, both of which were counteracted by the administration of probiotics. The permeability of the blood-brain barrier was increased in the model, but this effect was reversed by the probiotics for both brain regions. The gut barrier was permeated with the model, and this effect was reversed and dropped to lower levels than the control group after the administration of probiotics. Finally, lipid peroxidation showed a pattern of differences similar to that of permeabilities. The inhibition of the permeability of the blood-brain and gut barriers mediated by the administration of probiotics will likely provide protection by downregulating oxidative stress, thus affecting the rotarod test performance.

## 1. Introduction

Parkinson's disease (PD) is a neurodegenerative disorder characterized by the selective death of dopaminergic neurons of the substantia nigra pars compacta (SN*pc*), leading to denervation in the striatum (STR) and consequently a decrease in dopamine (DA) levels. This results in a loss of movement control and other nonmotor symptoms, such as cognitive impairment and sleep disorders, and even contributes to other diseases such as depression [1]. Some of these nonmotor symptoms include autonomic dysfunction and gut-related problems, such as constipation, which is very prevalent. The disease has been associated with dysfunction in the enteric nervous system and gut microbiota imbalance [2]. Although the pathogenesis of this disease remains unclear, experimental evidence shows that mitochondrial dysfunction and oxidative stress result from the formation of reactive oxygen species (ROS), neuroinflammatory processes accompanied by a decrease in anti-inflammatory cytokines, and low levels of antioxidant enzymes. These factors can alter the permeability of the blood-brain barrier (BBB) and the gut barrier, which are essential characteristics of the disease [3].

Regarding permeability of the former, experimental evidence shows that treatment with 1-methyl-4-phenyl-1,2,3,6-tetrahydropyridine (MPTP)—a neurotoxin that causes selective death of dopaminergic cells—increases the permeability of the BBB, probably through a decrease in the expression of the TJ: zonula occludens (ZO-1) and occludin in the STR [4–6]. On the other hand, comparative studies on the intestinal microbiota between patients with PD and healthy individuals reveal significant changes in the intestinal microbiota of PD patients. The intestinal microbiota of PD patients is characterized by a decrease in taxonomic diversity as well as significant differences in the representation of 9 genes and 15 species of microorganisms. This microbiological pattern can trigger local inflammation followed by *α*-synuclein aggregation and the formation of Lewy bodies [7]. This dysbiosis is linked to aberrant intestinal and systemic immune responses often accompanied by the production of inflammatory cytokines. Studies conducted by different research groups show that dysbiosis promotes motor deficits and neurodegeneration by activating neuroinflammation in mouse models with *α*-synuclein overexpression, all of which is caused by damage to the mucosa or gut barrier [8–10]. It is now clear that the pathology of PD includes an imbalanced gut microbiome. Therefore, a treatment that repairs the microbiome and, above all, inhibits the permeability of the gut barrier could improve some PD symptoms. Various probiotic treatments have been shown to reduce the decrease in high-sensitivity C-reactive protein and malondialdehyde (MDA) and increase glutathione levels, accompanied by the inhibition of ROS and inflammatory cytokine levels in PD patients [11]. In addition, the work conducted by Srivastav et al. [12] demonstrated that supplementation with a probiotic cocktail containing *Lactobacillus rhamnosus* GG, *Bifidobacterium animalis lactis*, and *Lactobacillus acidophilus* inhibited dopaminergic cell death in SN*pc* induced by MPTP and rotenone. This inhibition was accompanied by an increase in the expression levels of brain-derived neurotrophic factor (BDNF) and glial cell line-derived neurotrophic factor (GDNF) [11]. On the other hand, in a model of parkinsonism induction using 6-hydroxydopamine (6-OHDA), the probiotic mixture contains the bacterial strains *Streptococcus thermophilus*, *Bifidobacterium longum*, *Bifidobacterium breve*, *Bifidobacterium infantis*, *Lactobacillus acidophilus*, *Lactobacillus plantarum*, *Lactobacillus paracasei*, and *Lactobacillus delbrueckii* subsp. *bulgaricus*, and *Lactobacillus brevis* (formulation SLAB51) promoted a neuroprotective effect by inhibiting dopaminergic cell death. This effect was mediated by the activation of the peroxisome proliferator-activated receptor gamma (PPAR*γ*) derived from the production of short-chain fatty acids [12]. However, it is currently unknown whether oral administration of a probiotic cocktail (LLH135) containing *Lactobacillus fermentum* LH01, *Lactobacillus reuteri* LH03, and *Lactobacillus plantarum* LH05 has a neuroprotective effect on inhibiting the disruption of the BBB and the gut barrier in an induction model of hemiparkinsonism.

## 2. Materials and Methods

The experimentation was approved by the Comité Local de Ética en Investigación en Salud 1305 (Local Committee of Ethics in Health Research) under the institutional record number R-2023-1305-035, chaired by Dr. Blanca Miriam de Guadalupe Torres Mendoza, on May 31, 2023.

### 2.1. Probiotic LLH135 Production

*Lactobacillus fermentum* LH01, *Lactobacillus reuteri* LH03, and *Lactobacillus plantarum* LH05 were sourced from human milk and belong to the strain collection of the Industrial Microbiology Laboratory of CUCEI at the Universidad de Guadalajara. The strains were cultured in de Man Rogosa Sharpe medium (Cat. 288130; Difco) supplemented with 10% Nutraflora (Ingredion), incubated at 37°C for 24 hours, and harvested via centrifugation at 4500 rpm for 10 minutes, followed by washing with sterile physiological saline. The bacteria were suspended in sterile physiological saline and adjusted to a concentration of 10^9^ CFU/mL for oral administration. The suspension was kept at freezing temperature until use [13].

### 2.2. Animals and Experimental Design

The study was conducted using healthy male C57BL/6 mice weighing between 23 and 27 grams (*n* = 72) obtained from the Centro de Investigación Biomédica de Occidente (Western Biomedical Research Center). The mice were housed under controlled environmental conditions with a constant temperature of 23 ± 2°C and a 12:00 h light-dark cycle. They had access to food and water *ad libitum*, and all animal care and experimental procedures were performed following the official Mexican guidelines, NOM-062-ZOO-1999.

**Figure 1:**
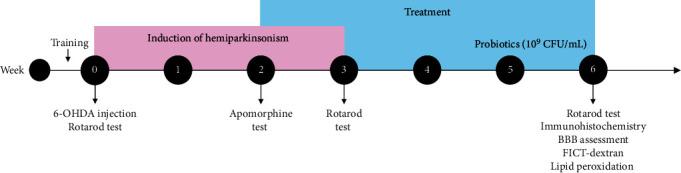
Experimental design.

The mice were randomly divided into four groups of 18 mice each: (1) Sham group, which was injured with 2 *μ*L of the 6-OHDA vehicle (0.1% ascorbic acid) (Cat. A-5960; Sigma-Aldrich) and received an oral gavage of physiological saline; (2) 6-OHDA group, which received an intrastriatal injection of 10 *μ*g/2 *μ*L of 6-OHDA; (3) LLH135 group, which received oral administration 100 *μ*L of a probiotic mixture consisting of *Lactobacillus fermentum* LH01, *Lactobacillus reuteri* LH03, and *Lactobacillus plantarum* LH05 at a concentration of 10^9^ CFU/mL for four weeks; and (4) 6-OHDA + LLH135 group, which received a combination treatment of 6-OHDA and oral administration of the probiotic mixture for four weeks (Figure 1).

### 2.3. Unilateral 6-OHDA Injection

The Parkinson's disease model was induced by the unilateral injection of 6-OHDA (Cat H4381; Sigma-Aldrich) into the STR of mice. The mice were anesthetized with a 4% induction dose and a 1-2% maintenance dose of isoflurane (Fluriso: VetOne 99%) and fixed onto a stereotaxic apparatus with their foreheads placed flat. A sagittal incision was made in the midline of the scalp to expose the skull. A 1 mm burr hole was drilled above the left STR at the following coordinates from bregma: anteroposterior (AP) +0.5 mm, mediolateral (ML) +2 mm, and dorsoventral (DV) -3.3 mm. Then, a 10 *μ*g dose of 6-OHDA (concentration of 5 *μ*g/*μ*L, final volume of 2 *μ*L as appropriate for the intracerebral ventricular route) was slowly administered at a rate of 1 *μ*L/min using a 10 *μ*L Hamilton syringe. After the infusion, the needle was kept stationary for 5 min at the injection site before being slowly removed. To protect noradrenergic neurons from 6-OHDA damage, an intraperitoneal injection of 25 mg/kg imipramine (Cat. 28.626-5; Sigma-Aldrich) was administered 30 min before the 6-OHDA infusion [14].

### 2.4. Apomorphine-Induced Rotation Test

To assess the variability of the unilateral 6-OHDA-induced PD mice, the apomorphine-induced rotation test was conducted 2 weeks after the lesions were made. A dopamine receptor agonist, apomorphine (1 mg/kg in 0.9% NaCl and 1% ascorbic acid) (Cat. 314-19-2; Tocris), was subcutaneously injected to trigger contralateral rotation. The contralateral rotations were recorded for 25 minutes; these rotations reflect the loss of dopaminergic neurons.

### 2.5. Rotarod Test

To evaluate the coordinated movement and balance of the mice, the rotarod test was conducted. The rotarod was equipped with automatic timers and drop sensors. Before the experiment, the mice were trained once a day for 3 days at a constant acceleration of 5 rpm/3 min. During the test, the mice were placed on the equipment, and the speed was slowly increased from 4 to 40 rpm over 5 min. The test was repeated three times with a rest interval of 15 min. The variable of interest was the fall latency, which was measured as the time elapsed from the moment the mouse was placed on the rotarod until it fell off. The evaluation was carried out before the injury and was repeated at 3 and 6 weeks [15].

### 2.6. Immunohistochemistry

After the last rotarod test, six animals from each group were anesthetized using pentobarbital sodium (100 mg/kg) and transcardially perfused with phosphate-buffered saline (PBS), followed by fixation using 4% paraformaldehyde. Brains were surgically extracted and sliced coronally to include the STR and SN*pc*. The presence of tyrosine hydroxylase (TH), which serves as a specific marker for dopaminergic neurons, was evaluated using immunohistochemistry staining. Free-floating slides were used, and cell membranes were permeabilized by incubating them for 20 minutes at 37°C with 0.3% sodium citrate. Nonspecific antigens were blocked with a solution consisting of 0.5% Triton X-100 in 1X PBS and 10% goat serum for 45 minutes under constant agitation and then washed with PBS three times for 5 minutes. Primary antibodies for TH (1 : 500, Cat. PA5-85167, Invitrogen) were added overnight at 4°C under constant agitation. After incubation, primary antibodies were removed, and tissues were washed with PBS and incubated with horse anti-mouse/rabbit secondary antibodies (1 : 1000, Cat. 30092; Vector Laboratories) for 2 hours at room temperature under dark conditions. The tissues were washed again with 1X PBS and then incubated for 2 hours with a matured avidin-biotin solution (Cat. PK6100; Vector Laboratories). Immunoreactivity was revealed using diaminobenzidine (DAB) (Cat. D5905; Sigma). The tissues were analyzed using a microscope, and microphotographs of the areas with immunoreactivity were taken and later quantified using the ImageJ 8 software [16]. Quantification of optical densities was obtained by subtracting background signals from negative control tissues which were processed the same way as the others but without the primary antibody. Mean intensities of pixels/area were compared after background subtraction. For morphological analysis of the STR and SN*pc*, coronal slices were cut at the bregma and −3.3 mm from the bregma, respectively, according to Paxinos and Franklin Atlas (2013) [17]. We collected ten sets of three tissue slices each from every brain processed for both regions; each slice was 30 *μ*m wide and was at least 200 *μ*m apart from another to avoid overcounting of cellular bodies in the SN*pc*. For the STR region, we preferred to select slices at the same coronal level in all mice to achieve the most uniform analysis.

### 2.7. BBB Assessment

Fluid-phase BBB permeability was assessed using sodium fluorescein (SF) at a 1-day post of finishing the treatments. Another set of mice (*n* = 6/group) were injected with 10% SF diluted in a saline solution at a dose of 2.5 mL/kg via i.p. (Cat. F6377; Sigma-Aldrich). Subsequently, 30 min after systemic fluorescein diffusion, the animals were anesthetized using a lethal dose of sodium pentobarbital (65 mg/mL; Pisa), and cardiac blood was collected, followed by transcardial perfusion with 15 mL of heparin (1,000 U/L) in PBS. The brains were removed, and SN*pc* and STR were dissected and weighed. To determine BBB permeability, the tissue was homogenized in 1/10 dilution in PBS and centrifuged at 16,060 × g for 15 min at 4°C; the supernatants were recovered and mixed with 95% ethanol to precipitate residual proteins and stabilize the fluorescent signal. SF fluorescence was measured in a fluorescent plate reader (Ex: 485 nm; Em: 525 nm) [18, 19].

### 2.8. FITC-Dextran

To evaluate barrier function, the remaining mice (*n* = 6/group) were fluorescently labeled, and dextran was used to measure gut barrier permeability. Following an overnight fast, mice were orally administered with a 100 mg/mL solution of 4 kDa fluorescein isothiocyanate-labeled dextran (FD4) (SKU 46944, Sigma) at a dose of 44 mg/100 g. Serum samples were collected four hours after administration by decapitating the animals. The concentration of serum FD4 was determined using the Synergy HT fluorimeter (BioTek) with excitation at 485 nm and emission at 530 nm and compared to known standards using serial dilutions [20].

### 2.9. Lipid Peroxidation

The tissues mentioned above were weighted and sonicated in a lysis buffer and protease inhibitor cocktail on an ice bath in a proportion of 100 microliters for each 10 mg of tissue. Then, homogenates were incubated on ice for 30 min and centrifuged at 10,000 × g for 30 min at 4°C. These samples were used to measure lipid peroxidation assessed by levels of MDA, which reacts with thiobarbituric acid (TBA). This reaction produces a colorful compound proportional to the levels of MDA with a peak of absorption at 540 nm, which was measured in a Multiskan GO Plate reader according to the parameters of the TBARS assay kit (item no. 10009055 Cayman) [21].

### 2.10. Statistical Analysis

The Kolmogorov test was realized to verify the normality of the data. Data that failed the test were analyzed employing nonparametrical methods (the two-stage linear step-up procedure of Benjamini, Krieger, and Yekutieli for the SF and FITC-dextran methods and the Kruskal-Wallis test for the TBARS assay). The data that presented normality were analyzed by parametrical methods (one-way ANOVA with a Tukey's post-hoc test for rotarod results and immunoreactivity for TH and an unpaired *T*-test for apomorphine). The significance level was set at *p* ≤ 0.05. The analysis was performed in the GraphPad 8.0.1 software.

## 3. Results

**Figure 2:**
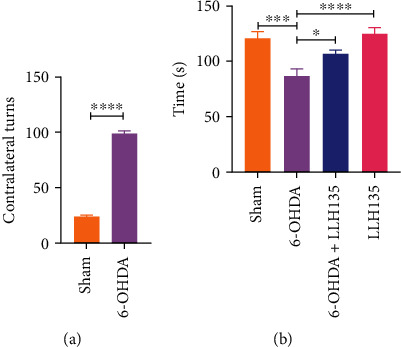
Apomorphine and rotarod tests. Contralateral turns in the apomorphine-induced rotation after injection of 10 *μ*g 6-OHDA (*n* = 72 mice total) (a). Evaluation of motor function in the different work groups after 4 weeks of treatment (b). Endurance performance in the rotarod test (*n* = 6 mice per group). Data are expressed as means ± SEM. ^∗^*p* < 0.05, ^∗∗∗^*p* < 0.001, and ^∗∗∗∗^*p* < 0.0001.

A commonly used indirect method to assess the decrease in dopamine levels and the extent of dopaminergic damage after 6-OHDA administration is the apomorphine-induced rotation test. Therefore, it was employed to confirm a correct lesion in all mice that underwent surgery, 18 for each group to a total of 72 mice. Student's *t*-analysis revealed statistical differences in the number of apomorphine-induced rotations between the sham and 6-OHDA groups (Figure 2(a); *p* = 0.001). Animals that responded to apomorphine were used to carry out immunohistochemical determinations and assess the permeability of the blood-brain and gut barriers.

### 3.1. Supplementation of Probiotic LLH135 Inhibits 6-OHDA-Induced Defects in Motor Coordination

One of the study's objectives was to evaluate motor coordination through the rotarod test. We determined whether the administration of the probiotic LLH135 could recover the motor impairment induced by the administration of 6-OHDA. In the animals lesioned with 6-OHDA, a decrease in the time spent on the rotarod was observed at 86.89 ± 6.65 sec compared to that of the sham treatment (122.1 ± 5.56 sec) (Figure 2(b); *p* = 0.001). However, the supplementation of probiotic LLH135 for 4 weeks partially improved behavioral dysfunction, increasing the latency to fall to 107.4 ± 3.67 sec compared to animals lesioned with 6-OHDA (Figure 2(b); *p* = 0.0463), respectively.

### 3.2. Supplementation of Probiotic LLH135 Protects Nigrostriatal DA Neurons from 6-OHDA-Induced Neurotoxicity

**Figure 3:**
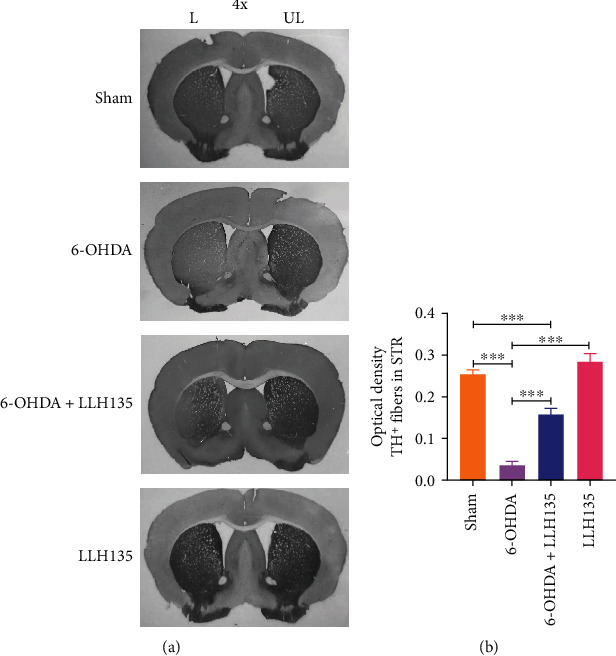
TH immunoreactivity in STR. Representative micrographs of immunohistochemistry (DAB staining) for TH^+^ in the STR after 4 weeks of treatment (4x magnification) (a). Evaluation of optical density TH^+^ fibers in STR (b). Data are expressed as means ± SEM (*n* = 6 mice per group). ^∗∗∗^*p* < 0.001.

**Figure 4:**
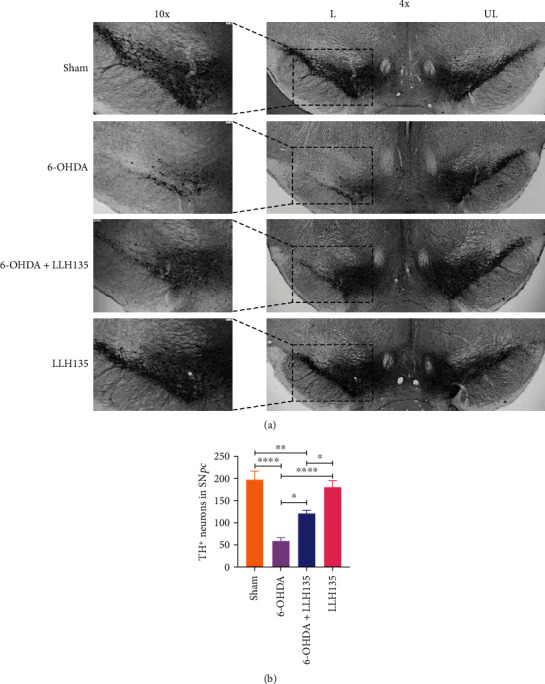
TH immunoreactivity in SN*pc*. Representative micrographs of immunohistochemistry for TH^+^ in the SN*pc* (a) after 4 weeks of treatment (4x and 10x magnifications). Evaluation of TH^+^ cell bodies in SN*pc* (b). Data are expressed as means ± SEM (*n* = 6 mice per group). ^∗^*p* < 0.05, ^∗∗^*p* < 0.01, and ^∗∗∗∗^*p* < 0.0001.

TH immunostaining revealed that 6-OHDA caused denervation in STR (Figure 3(a)) and selective death of dopaminergic neurons in SN*pc* (Figure 4(a)) compared to the sham control groups. Quantitative data confirmed that 6-OHDA induces dopaminergic neuronal death in SN*pc* (*p* = 0.0001) and loss of TH-positive dopaminergic fibers in STR (*p* = 0.0001), respectively, compared to the sham-treated mice. By contrast, mice treated with probiotic LLH135 exhibited a dramatic increase of TH-positive neurons in SN*pc* (Figure 4(b); *p* = 0.0154) and fibers in STR (Figure 3(b); *p* = 0.0001) compared to 6-OHDA-treated mice.

### 3.3. Supplementation of Probiotic LLH135 Inhibits the Permeability of the Blood-Brain and Gut Barriers from 6-OHDA-Induced Neurotoxicity

**Figure 5:**
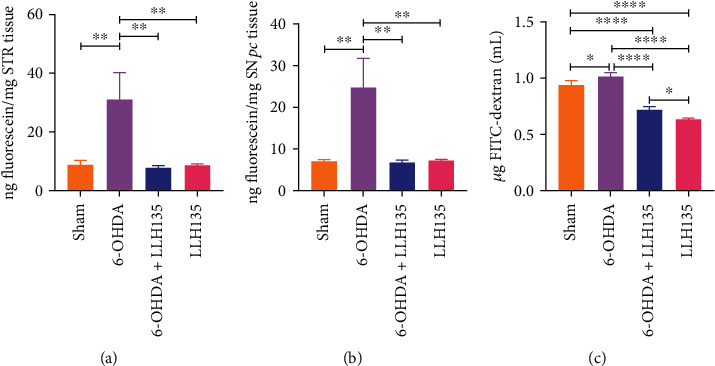
Treatment effects in permeability on gut and blood-brain barrier. BBB permeability was demonstrated by fluorescein extravasation in the STR (a) and SN*pc* (b). Gut permeability was determined by the serum levels of FITC-dextran (4 kDa) concentration measured 4 hours after oral administration (c). Data are expressed as means ± SEM (*n* = 6 mice per group). ^∗^*p* < 0.05, ^∗∗^*p* < 0.01, and ^∗∗∗∗^*p* < 0.0001.

BBB permeability was assessed by SF uptake in the SN*pc* and STR. SF uptake into SN*pc* and STR was significantly increased in the 6-OHDA group compared to the sham (Figures 5(a) and 5(b); *p* = 0.0044 and *p* = 0.0069, respectively). By contrast, mice treated with probiotic LLH135 in combination with 6-OHDA exhibited a dramatic decrease in SF uptake in both brain regions (Figures 5(a) and 5(b); *p* = 0.004 for STR and *p* = 0.0036 for SN*pc*).

Regarding the evaluation of the permeability of the gut barrier, it was carried out through the determination of the levels of FITC-dextran concentration in plasma. Compared to the sham mice, the administration of 6-OHDA showed increased FITC-dextran permeability from 0.9418 ± 0.02855 *μ*g/mL to 1.015 ± 0.02879 *μ*g/mL (Figure 5(c); *p* = 0.046), whereas mice treated with probiotic LLH135 in combination with 6-OHDA exhibited a dramatic decrease in FITC-dextran permeability from 1.015 ± 0.02879 *μ*g/mL to 0.7223 ± 0.02389 *μ*g/mL (Figure 5(c); *p* = 0.0097).

### 3.4. Supplementation of Probiotic LLH135 Inhibits Lipid Peroxidation from 6-OHDA-Induced Neurotoxicity

**Figure 6:**
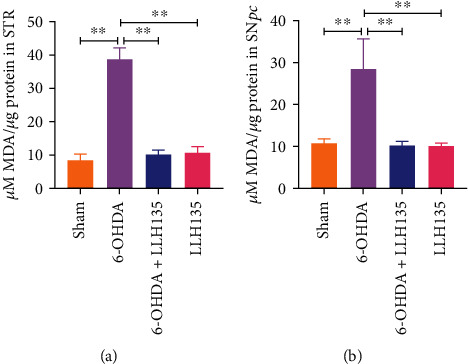
Lipid peroxidation was measured as MDA concentration. The thiobarbituric acid reactive substance (TBARS) assay was performed to determine MDA levels, and these were obtained from STR (a) and SN*pc* (b). Data are expressed as means ± SEM (*n* = 6 mice per group). ^∗∗^*p* < 0.01.

Mice subjected to 6-OHDA treatment showed a significant increase in MDA in the STR and SN*pc* compared to the sham group (*p* = 0.0007 and *p* = 0.0062, respectively) (Figure 6). These effects of 6-OHDA were reversed by the administration of probiotics (LLH135); therefore, the 6-OHDA + LLH135 group showed decreased MDA levels compared to 6-OHDA alone (*p* = 0.0062 and *p* = 0.0033) in both the STR and SN*pc*, respectively. Finally, in this trial, when only the probiotic LLH135 was administered to the mice, the MDA levels did not change compared to the sham group in both brain regions.

## 4. Discussion

In this study, we found that the 4-week supplementation of probiotic LLH135 (*Lactobacillus fermentum* LH01, *Lactobacillus reuteri* LH03, and *Lactobacillus plantarum* LH05) significantly prevented the loss of dopaminergic neurons, improved motor function in PD mice, decreased the permeability of the blood-brain and gut barriers, and reduced lipid peroxidation after 6-OHDA injury.

Various experimental evidence described in the literature suggest different pathways or mechanisms of action by which these probiotics could exert these neuroprotective effects. Castelli et al. describe two mechanisms. First, the inhibition of dopaminergic cell death is attributed to the supplementation of probiotics, prebiotics, and symbiotics, which increase energy metabolism in the brain through enhanced mitochondrial activity and glycolysis. The second proposed mechanism is through an increased production of short-chain fatty acids (SCFAs) and an enhanced antioxidant enzyme activity [22]. Mitochondrial dysfunction leads to increased oxidative stress through the generation of ROS, such as superoxide anion and hydrogen peroxide, which play an important role in the development of PD [23–26]. Studies in mice treated with MPTP demonstrated a decrease in the antioxidant capacity of superoxide dismutase (SOD) and glutathione (GSH) in the nigrostriatal pathway. The administration of *Lactobacillus plantarum* (PS128) restored the antioxidant capacity of these enzymes and increased the activity of catalase and glutathione peroxidase 1 (GPx) [27]. Additionally, work by Nurrahma et al. demonstrated that long-term supplementation with the probiotic AP-32 increased antioxidant capacity through its SOD and GPx activity in the serum of 6-OHDA-lesioned rats, suggesting that one of the mechanisms by which probiotics inhibit dopaminergic cell death caused by oxidative stress is through restoring the capacity of antioxidant enzymes in the CNS [16]. Another biomarker for the oxidative status that has been linked to the progression and presence of PD is the plasmatic levels of MDA [28]. This molecule has been described as essential for induced cell death and, along with 4-hydroxy-2-hexenal (HNE), serves as a reliable indicator of lipid peroxidative status in general [29, 30]. Consequently, our results are in line with reports of increased MDA levels in 6-OHDA *in vitro* and *in vivo* models [31].

Probiotics exert their neuroprotective actions primarily through the production of SCFAs. These acids (acetate, propionate, and butyrate) are produced in the gastrointestinal tract, particularly in the colon, through the fermentation of dietary fiber by the intestinal microbiota. They are used by the intestinal epithelium as an energy substrate to maintain its integrity and function. SCFAs are known to exert antioxidant properties, have immunomodulatory functions, and regulate energy metabolism [16, 32, 33]. It is worth noting that various experimental evidence have demonstrated decreased levels of propionic acid, butyric acid, and caproic acid in PD patients compared to healthy controls [2]. Additionally, a decrease in the abundance of *Prevotella* (propionate-producing) was observed in the feces of PD patients [34], and the abundance of putative butyrate-producing bacteria, such as *Faecalibacterium prausnitzii*, *Blautia*, *Coprococcus*, *Roseburia*, and *Eubacterium*, also decreased [35, 36]. Therefore, a decrease in the levels of these fatty acids in the serum may result from alterations in the intestinal microbiota and could impact the development of Parkinson's disease. In the work conducted by Shin et al., a correlation was observed in low levels of propionic acid with the Unified Parkinson's Disease Rating Scale (UPDRS) part III in patients with Parkinson's disease [37]. Propionic acid has been shown to inhibit neuroinflammatory activation and attenuate blood-brain barrier damage [38], which are two characteristic pathological changes in the PD brain [39].

On the other hand, it has been shown that butyric acid (200 mg/kg body weight) also exerts neuroprotective effects by inhibiting dopaminergic cell death in the SN*pc* and STR and improving motor symptoms in mice administered with MPTP. This mechanism is mediated through an increase in the expression of glucagon-like peptide 1 (GLP-1) in the colon, which could act as a neurotrophic factor. In addition, butyric acid (150 *μ*m) has been shown to inhibit dopaminergic cell death by inhibiting *α*-syn-induced damage to genetic material through the inhibition of various histone deacetylases and subsequent upregulation of DNA repair genes [40]. Furthermore, one of the mechanisms by which SCFAs exert their neuroprotective action is through the activation of their specific receptors (FFAR2/3), which are G protein-coupled receptors. Once these receptors are activated, intracellular signaling pathways are triggered, leading to the activation of the AMPK pathway. This, in turn, activates the Nrf2 factor (nuclear erythroid 2-related), causing the translocation to the nucleus and its binding to the ARE, thus promoting the gene expression of several antioxidant enzymes (heme oxigenase-1 (HO-1), quinone oxidoreductase 1 (NQ01), GPx, catalase, etc.), inhibiting the oxidative stress promoted by ROS and potentially inhibiting dopaminergic cell death after administration with 6-OHDA [41]. Previous studies in our laboratory have demonstrated that NQ01 activity decreased after a model of parkinsonism. Along with this, we observed increased immunoreactivity for 8-hydroxyguanosine as evidence of oxidative stress-mediated DNA damage. These effects were reversed in a treated group, likely through the activation of Nrf2 via another pathway in the same brain regions [42].

It is worth mentioning that the maintenance of intestinal health depends to a large extent on SCFA. Therefore, a deficiency of SCFA can result in impaired intestinal immunity and a loss of integrity in the intestinal epithelium [43]. This allows bacteria, lipopolysaccharides (LPS), and other toxic substances to enter the circulation, leading to systemic inflammation and the development of various health problems in different organs, including the brain. Butyrate, a crucial energy substrate for colonocytes, offers several additional benefits: it stimulates mucin secretion, which prevents LPS uptake, reduces inflammation in epithelial cells, increases the activity of antioxidant systems, and improves barrier integrity by increasing the expression of tight junction (TJ) proteins [44].

The decrease in TJ protein levels is a crucial process that leads to the opening of the BBB in pathological situations. Restoring the integrity of the BBB requires the recovery of TJ protein expression, which can be induced by various chemical factors, including SCFAs [45]. Pathological stimuli activate NF-*κ*B-mediated expression of multiple genes, including MMP-9 and NLRP3, which cause the degradation of TJ proteins. However, SCFA-promoted Nrf2 activation can counteract this inflammatory response. SCFAs can suppress NF-*κ*B and promote Nrf2 activation, leading to the restoration of TJ protein expression. SCFAs can enter cells through transporters such as MTC1 and FAT/CD36 or by binding to receptors such as GPR41 [46]. Once inside the cell, SCFAs inhibit histone deacetylase (HDAC), which leads to an increase in histone acetylation. This increase in acetylation facilitates the expression of genes, including Nrf2, that contribute to BBB integrity [47].

In models of parkinsonism induction, sodium butyrate has been shown to inhibit the permeability of the BBB through the upregulation of occludin and ZO-1. These effects were accompanied by improved neurobehavioral impairment, including cognitive abilities, motor coordination performance, and decreased nigrostriatal pathway dopaminergic cell death [48]. Additionally, SCFAs increase NF-*κ*B acetylation, which inhibits its transcriptional activity. Under pathological conditions, MLCK phosphorylates MLC2, which leads to the formation of actin stress fibers and the endocytosis of transmembrane TJ proteins, resulting in BBB breakdown. However, SCFAs can promote the reassembly of TJs by suppressing the MLCK/MLC2 pathway [45].

Inhibition of HDAC by SCFAs also leads to the hyperacetylation of PPAR*γ*, which facilitates its translocation into the nucleus, where it potentiates the transcription of TJPs [49, 50]. Furthermore, the administration of SCFAs promotes the activation of the Wnt signaling pathway and promotes the translocation of *β*-catenin into the nucleus, which upregulates the transcription of ZO-1, occludin, and claudin-5. The interaction between SCFA signaling and the canonical Wnt/*β*-catenin pathway is plausible [51, 52].

Overall, our findings suggest that the administration of a probiotic cocktail (LLH135) containing *Lactobacillus fermentum* LH01, *Lactobacillus reuteri* LH03, and *Lactobacillus plantarum* LH05 is a promising candidate for the prevention of dopaminergic neuronal damage and the permeability of the blood-brain and gut barriers. This confirms that the modulation of the intestinal microbiota plays an important role in the regulation of dopaminergic cell death in models of parkinsonism induction. All these effects could be reasonably attributed to the effects of SCFAs by the different mechanisms abovementioned. These SCFAs could be produced by the bacteria present in the probiotic mixture as well. However, one of the limitations of the present study was the lack of measurement of these SCFAs in the *in vivo* model. Another limitation is that we were not able to sequence samples from the microbiota. This information could have provided more knowledge about the ways in which the probiotics produced their effects.

## Data Availability

The data that support the findings of this study are available from the corresponding author, Flores-Soto ME, upon reasonable request.
